# The Use of *Ascophyllum nodosum* and *Bacillus subtilis* C-3102 in the Management of Canine Chronic Inflammatory Enteropathy: A Pilot Study

**DOI:** 10.3390/ani11123417

**Published:** 2021-11-30

**Authors:** Marco Isidori, Fabrizio Rueca, Francesca Romana Massacci, Manuela Diaferia, Andrea Giontella, Marco Caldin, Tommaso Furlanello, Ronald J. Corbee, Gabriele Mannucci, Giovanni Pezzotti, Massimo Trabalza-Marinucci

**Affiliations:** 1 Department of Veterinary Medicine, University of Perugia, Via San Costanzo 4, 06126 Perugia, Italy; marco.isidori2@studenti.unipg.it (M.I.); fabrizio.rueca@unipg.it (F.R.); manuela.diaferia@unipg.it (M.D.); giontella@gmail.com (A.G.); massimo.trabalzamarinucci@unipg.it (M.T.-M.); 2 Istituto Zooprofilattico Sperimentale Umbria e Marche ‘Togo Rosati’, Via G. Salvemini 1, 06126 Perugia, Italy; g.pezzotti@izsum.it; 3 Clinical Pathology Division, San Marco Veterinary Clinic and Laboratory, Via dell’Industria 3, 35030 Veggiano, Italy; mc@sanmarcovet.it (M.C.); tf@sanmarcovet.it (T.F.); 4 Department of Clinical Sciences of Companion Animals, Faculty of Veterinary Medicine, Yalelaan 108, 3584 CM Utrecht, The Netherlands; r.j.corbee@uu.nl; 5 Landini Giuntini S.p.A., Via Rosa Luxemburg 30, 06012 Città di Castello, Italy; gabriele.mannucci@landinigiuntini.it

**Keywords:** enteric diseases, seaweed, probiotic, microbiota, faecal metabolites

## Abstract

**Simple Summary:**

The development of novel, safe and effective therapies is highly desired for managing diseases requiring long-term use of drugs endowed with side effects. In the present investigation, we aimed to assess the impact of gut microbiota modulation strategies, i.e., the brown seaweed *Ascophyllum nodosum* and/or the probiotic microorganism *Bacillus subtilis* C-3102, in dogs diagnosed with Canine Chronic Inflammatory Enteropathy. The tested agents were administered on top of a hydrolysed protein diet, in a step-up fashion at 30-day intervals, with faecal collection and clinical evaluation of disease severity being performed before study initiation as well as at the end of each treatment phase. Neither *Ascophyllum nodosum*, nor its association with *Bacillus subtilis* C-3102, were able to significantly alleviate clinical signs and augment bacterial richness within the study population. Notwithstanding, increased numbers of beneficial carbohydrate-fermenting bacteria, alongside with higher amounts of faecal acetate were recorded, suggesting a potentially positive effect on gut health.

**Abstract:**

The aim was to assess the effects of *Ascophyllum nodosum* (AN) with/without *Bacillus subtilis* C-3102 as alternative treatments for Chronic Inflammatory Enteropathy (CIE) of dogs. Fourteen CIE patients, which had received the same control (CTR) diet, were enrolled to serially receive three diets: (1) hydrolysed protein (HP) diet; (2) 4.0% AN supplemented HP (HPA) food, (3) HPA diet fortified with 125 billion *B. subtilis* C-3102 spores/10 kg body weight (HPAB diet). Clinical outcome was assessed by Canine Inflammatory Bowel Disease Activity Index (CIBDAI), whereas gut microbiota compositional variations were investigated via 16S rRNA gene analysis, and faecal fermentation end-products by liquid chromatography. Higher abundances of the *Ruminococcaceae* and *Rikenellaceae* families were shown in HPA relative to CTR treatment, with *Bacillus* genus being differentially abundant on HPAB diet. Concentrations of acetate were higher (*p* < 0.05) in dogs fed HPA compared to CTR diet, and amounts of isovalerate and isobutyrate were greater (*p* < 0.05) in HPA compared to HP food. A tendency for higher amounts of faecal butyrate was found for the HPAB treatment (*p* = 0.06). Comprehensively, while displaying potentially positive effects on faecal fermentations, the tested substances failed to improve CIBDAI scores and microbial richness in CIE dogs.

## 1. Introduction

Chronic inflammatory enteropathy (CIE) is a collective term used to describe a group of chronic inflammatory disorders of the canine gastrointestinal (GI) tract, for which diagnosis is made by ruling out other identifiable causes of vomiting and/or diarrhoea [1]. Clinically, three different phenotypes can be recognised in retrospection based on treatment response, i.e., food-responsive enteropathy (FRE), antibiotic-responsive enteropathy (ARE), and immune-suppressant-responsive enteropathy (IRE) [2]. While the exact nosological classification currently remains unsettled, the different forms bear resemblance in terms of clinical features, endoscopic appearance, and histological lesions [3]. Besides, a large body of studies harnessing omics-based tools have documented an association between CIE and intestinal dysbiosis (ID), the latter being defined as an imbalance of the composition and metabolic capacity of the gut microbiota [4]. Regardless of whether ID is an inciting cause or an effect of the intestinal disease [5], it becomes an integral component of the CIE pathophysiology itself by undermining the complex yet delicate balance between the host’s immune system and the intestinal symbionts, eventually leading to a pro-inflammatory milieu [6]. As such, manipulation strategies of the intestinal microbiota may serve as sound therapeutic agents to either cure or palliate CIE. Most extreme examples of microbial-targeted interventions with proven clinical efficacy are represented by antibiotics [7]. In spite of the well-established therapeutic value, the indiscriminate administration of antimicrobials in CIE has been disputed in recent times, due to the deleterious consequences they may have for both the animal’s health and the general public [8,9]. Conversely, more gentle approaches for gut microbiota modulation, such as the use of prebiotics, probiotics, and faecal transplant are currently under investigation as promising alternatives to antibacterial treatment [10].

*Ascophyllum nodosum* (AN), commonly known as kelp or egg wrack, is a brown marine alga thriving along the rocky, temperate shores throughout the North Atlantic area [11]. Aside from its being an important bio-resource of essential nutrients [12], AN contains a large amount of complex glycans such as alginates, fucoidans, and laminaran [13] as well as secondary metabolites, like phlorotannins [14]. These biopolymers account for a wide variety of biological activities, ranging from anti-inflammatory to prebiotic actions [15,16,17]. A clinical trial revealed that edible treats containing AN efficiently decreased plaque and calculus accumulation in client-owned dogs with naturally occurring periodontal disease after professional dental treatment [18]. According to a recent study, AN palatability in extruded dog foods has been regarded as acceptable [19]. However, despite the relatively high diffusion in pet food, other clinical investigations evaluating effects of AN in canines are currently lacking. It can be hypothesised that AN also has beneficial effects in CIE, as has been suggested by in vitro and in vivo murine studies of inflammatory intestinal diseases [20].

*Bacillus subtilis* C-3102 is a rod-shaped, Gram-positive, sporulating bacterial strain recently authorised as “gut flora stabiliser” in dogs by the European Commission [21]. The spore-forming ability of *B. subtilis* C-3102 imparts it several advantages over traditional probiotic micro-organisms. Indeed, metabolic dormancy coupled with resistance properties of endospores allow for longer storage periods and improved viability during gut transit [22]. Besides, strain *B. subtilis* C-3102 is able to withstand different industrial technologies (e.g., heat and high-pressure treatment), which favours its use in canine food production [23,24]. While being regarded as allochthonous to the animal’s GI tract [25], *B. subtilis* C-3102 is endowed with attractive bio-functions towards intestinal health. Recorded beneficial effects associated with *B. subtilis* C-3102 strain administration in healthy dogs entail improvement in faecal quality, enhancement of fat and carbohydrate digestibility, reduction of faecal ammonia, and accretion of faecal short chain fatty acids (SCFA) concentrations, as well as increase in gut bacterial diversity [26,27,28]. However, except for isolated reports [29], its impact on chronic inflammatory GI disorders of dogs still remains largely unexplored.

In view of the foregoing considerations, the present study was envisioned to evaluate the effects of AN, with or without *B. subtilis* C-3102 administration, on clinical outcome, faecal microbiota composition, and faecal fermentation products in CIE cases not responding to dietary manipulations.

## 2. Materials and Methods

### 2.1. Inclusion and Exclusion Criteria of Animals

Selection of patients with CIE was performed at a public canine shelter following a diagnostic exclusion protocol, according to international guidelines [30] (Figure S1). Medical records of the canine shelter population (n = 390) were scrutinised for a history of chronic GI signs, i.e., vomiting and/or diarrhoea enduring for 3 or more weeks, with or without weight loss. A total of 115 dogs, which fulfilled the anamnestic inclusion criteria, were screened for geohelminth eggs via centrifugal faecal flotation with zinc sulphate solution [31], as well as for *Giardia duodenalis* cysts and *Cryptosporidium* spp. oocysts by immunofluorescence assays [32]. Patients testing positive for parasitic infestations (n = 77) were excluded from further diagnostic work-up, being impossible to carry out proper disinfestation operations within the shelter. Faecal culture to detect pathogenic bacteria (e.g., *Salmonella* spp., *Campylobacter* spp. and *Clostridium perfringens*) was not performed due to the low diagnostic value in diarrhoeic dogs [33].

The remaining animals (n = 38) underwent a 6-week dietary trial based on an ad hoc formulated hydrolysed dry food (Landini Giuntini S.p.A., Città di Castello, Italy), to attempt to diagnose FRE cases. Diet composition, along with results of chemical analyses are displayed in Table 1. It was decided to leave out food-responders (n = 24) from the present study as dietary therapy alone is known to have a considerable effect on clinical, histological, and microbiological outcomes in patients affected by this disease sub-type [34,35,36]. Conversely, dogs failing to respond to dietary challenge (n = 14) were subject to a thorough physical examination, blood works (i.e., complete blood count, serum biochemistry profile, serum proteins electrophoresis, serum folate and cobalamin concentrations), as well as serum trypsin-like immunoreactivity concentration, to exclude other causes of chronic GI signs. Additional assays, such as serum basal cortisol or ACTH stimulation test, were performed on a case-specific basis. Thereafter, a two-week course of oral tylosin (25 mg/kg q24) [37] was initiated to diagnose ARE. Ultimately, canine patients whose clinical signs persisted after antibiotic treatment (n = 4), underwent coagulation profile testing, abdominal ultrasound, and gastro-duodenoscopy with histopathological analysis of endoscopically procured biopsies to issue an exclusion diagnosis of IRE.

### 2.2. Study Design

A 90-day longitudinal, non-comparative investigation was carried out to evaluate the effects of AN with or without *B. subtilis* C-3102 supplementation on clinical, microbiological and faecal fermentative parameters in the canine patients formerly diagnosed with either ARE or IRE. The animals were individually housed in indoor enclosures with a concrete floor, measuring 6 square meters, and with access to shared gravel runs. Each cage was equipped with a sleeping mat as well as an automatic waterer supplying fresh tap water. Animal well-being was promoted through environmental enrichment (i.e., food dispensing toys) and by walking the dogs daily in outdoor exercise areas. A highly digestible extruded dog food, hereinafter referred to as control diet (CTR), was fed to all the patients for three months before entering the study.

Three different dietary treatments were administered to the study population in a step-up fashion at 30-day intervals, with no wash-out period being interposed between two consecutive phases. Treatments were as follows: (1) hydrolysed animal protein (HP) diet, the same diet used to diagnose FRE cases (see Table 1); (2) AN-supplemented HP diet (HPA), obtained by replacing a 4.0% protein pea seeds with an equal amount of powdered anhydrous AN (Tasco^®^; Acadian Seaplants Limited, Dartmouth, NS, Canada) to the formula of the HP diet during the manufacturing process; (3) *B*. *subtilis* C-3102-supplemented HPA diet (HPAB), obtained by mixing HPA with *B. subtilis* C-3102 (Calsporin^®^; Asahi Calpis Wellness Co., Ltd., Tokyo, Japan) at a dosage of 125 billion (125 × 10^9^) lyophilised bacteria per 10 kg body weight, prior to meal feeding. Fortification levels of AN as well as *B. subtilis* C-3102 were either extrapolated from reports in other animal species or based on the manufacturers’ suggestions [38,39].

**Figure 1:**
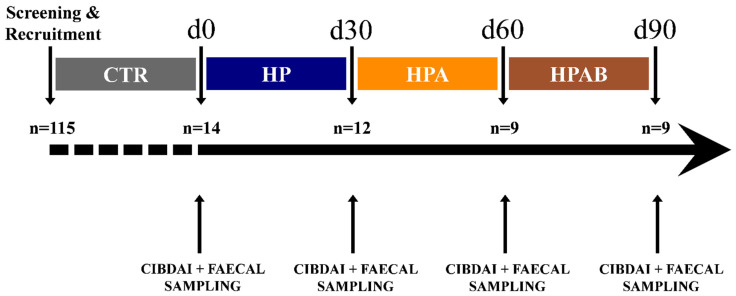
Study timeline and flow of dogs with chronic inflammatory enteropathy. CTR: control diet; HP: hydrolysed protein diet; HPA: *Ascophyllum nodosum* supplemented HP diet; HPAB: *Bacillus subtilis* C-3102 supplemented HPA.

Throughout the study, the patients did not receive any other additional foods or pharmacological treatments. Canine IBD activity index (CIBDAI) [40] evaluation, along with faeces collection, were performed at four distinct time points, i.e., prior to study initiation (d0), at its end (d90), and right before each treatment switch (d30, d60), as summarised in Figure 1. Freshly voided stool specimens were aliquoted into two parts, whereof one was immediately suspended in DNA/RNA Shield™ solution (Zymo Research, Irvine, CA, USA) for microbiota analysis and the other collected into air-tight disposable containers (Aptaca, Canelli, Italy) for faecal biochemistry assays. Faecal samples were transported at refrigeration temperature (+4 °C) to the laboratory and frozen at −80 °C until analysed.

### 2.3. Chemical Analyses of Foods

HP was chemically analysed according to the Association of the Official Analytical Chemists [41] to determine the dry matter (method 934.01), ether extracts after acid hydrolysis (method 954.02), ash (method 942.05), crude fibre (method 962.09), total dietary fibre (TDF; method 991.43), and crude protein (method 954.01). Van Soest fibre fractions (i.e., neutral detergent fibre, acid detergent fibre and acid detergent lignin) were assayed following methods of Van Soest et al. [42]. Metabolisable energy content (ME) of the food was calculated in accordance with the predictive equation based on digestible energy estimated as a function of crude fibre (CF) [43].

### 2.4. Faecal Metabolite Analyses

All faecal samples were shipped in dry ice to the same laboratory (Laboratorio d’Analisi Veterinarie San Marco, Padova, Italy). Faecal concentrations of linear (L)-SCFA (i.e., formic acid, acetic acid, propionic acid and butyric acid), acetone, lactic acid, branched (B)-SCFA; (i.e., isovaleric acid, isobutyric acid, and valeric acid), phenolic and indolic compounds (i.e., skatole, indole and phenol) were measured according to an adapted version of previously described protocols [44,45].

First, primary stock solutions (400 µg/mL) were prepared by dissolving the compounds in a mix solution of water and acetonitrile (60:40 *v*/*v*) for standard analyses. Diluted working standard solutions were prepared from these stock standards and used to construct the calibration curves as well as to create quality control samples. All solutions were stored at −20 °C and used within two months of preparation.

An amount of 500 μL of water with trifluoroacetic acid (0.1% *v*/*v*) was added to each faecal sample. After vortexing and centrifuging the mixture at 64,000× *g* for 20 min at +4 °C, the supernatant was diluted and filtered through a 0.2 µm pore-sized syringe filter (PTFE hydrophilic, Whatman, UK). Then, 20 μL of the filtered solution was injected in the liquid chromatography system (Acquity UPLC System, Waters Corporation, Manchester UK) equipped with a cooling auto-sampler and column oven, enabling temperature control of the analytical column. An Acquity UPLC HSS C18 column (2.1 × 150 mm, 1.8 μm; Waters Corporation) was employed for separation, maintaining the column temperature at +37 °C. The chromatographic flow-rate was set at 0.8 mL/min, with a gradient elution of a mobile phase comprising two eluents, respectively solvent A (water with 0.1% *v*/*v* trifluoroacetic acid) and solvent B (pure acetonitrile). Total analysis run time was 15 min. A solution of water-acetonitrile (90:10 *v*/*v*) was used as strong wash and weak wash solvent to avoid any carry-over from previous injections. A partial loop with needle overfill was selected as the injection mode, with an injection volume of 5 μL. The temperature of the auto-sampler was maintained at +10 °C. Analytes quantification was achieved via diode-array detection. All data collected in centroid mode were processed using MassLynx 4.1 software with targetLynx program (Water Corp., Milford, MA, USA). The method was validated according to the US Food and Drug Administration guidelines for bioanalytical method validation [46]. The method was validated for the selectivity, linearity, precision, accuracy, recovery, and stability. Selectivity and sensitivity were assessed by comparing the chromatogram of blank faeces with those of corresponding faecal samples spiked with analytes’ standards.

### 2.5. Faecal DNA Extraction and 16S rRNA Gene Sequencing

From each faecal aliquot, total DNA was extracted using the QIAamp PowerFecal DNA Kit (Qiagen, Valencia, CA, USA), according to the manufacturer instructions.

Microbial profiling was performed using high-throughput sequencing of the V3-V4 hypervariable region of the 16S rRNA gene (2 × 300 bp paired-end reads) on an Illumina MiSeq platform following the standard Illumina sequencing protocol and by using primers PCR1F_343 (5′-CTTTCCCTACACGACGCTCTTCCGATCTACGGRAGGCAGCAG-3′) and PCR1R_784 (5′-GGAGTTCAGACGTGTGCTCTTCCGATCTTACCAGGGTATCTAATCCT-3′).

Quality control was performed on the resulting FastQ files using FastQC software (https://www.bioinformatics.babraham.ac.uk/projects/fastqc/, accessed on: 11 January 2021); the files were then analysed using QIIME2 tool (version 2019.4.) [47] and the subsampled open-reference OTU picking process [48]. The Illumina adapters were removed through the ‘cutadapt’ function. Subsequently, the sequences were clustered into an Operational Taxonomic Unit (OTU) against the SILVA 132 ribosomal RNA (rRNA) databases [49]. Singleton OTUs and OTUs representing less than 0.005% of the total number of sequences were removed from the dataset as recommended by the QIIME software authors [50]. Samples with less than 10,000 reads after quality control procedures were eliminated, which resulted in excluding from the analysis one sample (sample ID: 480956F453218).

### 2.6. Biostatistical Analyses

Statistical analyses were performed in R (v. 4.0.3) [51] using a linear mixed-effects model (LMEM). The best-fit model was identified using the Akaike’s information criterion. The effects of dietary treatment and age on clinical outcome and faecal metabolite concentrations were tested using a model where dog was modelled as a random effect to account for repeated measures for individual dogs; treatment (CTR, HP, HPA, HPAB diets) and age (group 1 ≤ 5 years vs. group 2 ≥ 6) were modelled as fixed, categorical variables. Clinical phenotype (ARE vs. IRE) effect was not included in the model because it was found to be not significant. If the effect of treatment or age was significant, Tukey’s test was used for post-hoc analysis. Statistical significance and tendency were set at *p* ≤ 0.05 and 0.05 < *p* < 0.1, respectively.

For the microbiota composition analysis, the BIOM OTU table was imported into R (v. 4.0.3; www.r-project.org, accessed on: 11 January 2021) [51] using Phyloseq package (v. 1.34.0) [52]. The vegan (v. 2.5-7) package [53] was used to perform rarefaction analyses of the OTUs in the whole dataset. Richness and diversity analyses were performed at the OTU level. The α-diversity was calculated using the Shannon index and the β-diversity using the ‘betadisper’ function of the Vegan R package for the Whittaker’s index. The ANOVA (‘aov’ function) was performed in order to assess differences for the α-diversity, β-diversity, and log-transformed richness. Moreover, the post-hoc comparisons were performed using the Tukey’s Honest Significant Differences (HSD) test.

The non-metric multidimensional scaling (NMDS) by using Bray–Curtis dissimilarity values was performed in order to evaluate differences in the overall composition of faecal microbiota. To identify the statistical significance of the study variables within the NMDS ordination space, the ‘env_fit’ function was used. Permutational multivariate analyses of variance (PERMANOVA) were performed using the ‘adonis’ function. The significance threshold was set at *p* < 0.05.

The OTU differential abundance (DA) analysis was performed using the metagenomeSeq package (v. 1.32.0) [54] by including the raw OTU counts prior to rarefaction. The OTU counts were normalised using the cumulative sum scaling method, and a zero-inflated Gaussian distribution mixture model (‘fitZig’ function) was employed to assess differences in relative OTU abundance. The significance level was set to a false discovery rate (FDR) lower than 0.05.

## 3. Results

### 3.1. Clinical Outcome and Faecal Fermentative Products

A total of 14 sheltered dogs, which had received a final diagnosis of either ARE (n = 10) or IRE (n = 4) at the end of the diagnostic exclusion protocol, were selected for the investigation. At the time of enrolment all the animals were symptomatic, including antibiotic-responsive cases, which had a foreseeable recurrence of symptomatology shortly after the end of antimicrobial therapy [2]. Diarrhoea was the most common clinical sign observed, being prevalently mixed or of large bowel origin. Breeds included crossbreed (n = 8), Italian Corso Dog (n = 2), and 1 dog each of the following: American Pit Bull Terrier, American Bulldog, American Staffordshire Terrier, and Rottweiler. Patients had a mean age of 5.1 ± 2.1 (2–8) years, mean body weight of 29.1 ± 6.4 (16.6–40.6) kilograms, and a body condition score of 4.7 ± 1.0 (3–7) out of 9. Ten dogs were male and four were female, all of which were castrated/neutered.

Of the 14 dogs enrolled, only nine completed the investigation. Dropout reasons were: adoption (n = 2), worsening of clinical conditions (n = 2), and concurrent illness (i.e., urolithiasis; n = 1). Only data from patients that did complete the study were used for statistical analyses (Table S1). A statistical summary of the investigated parameters is provided in Table S2.

**Figure 2:**
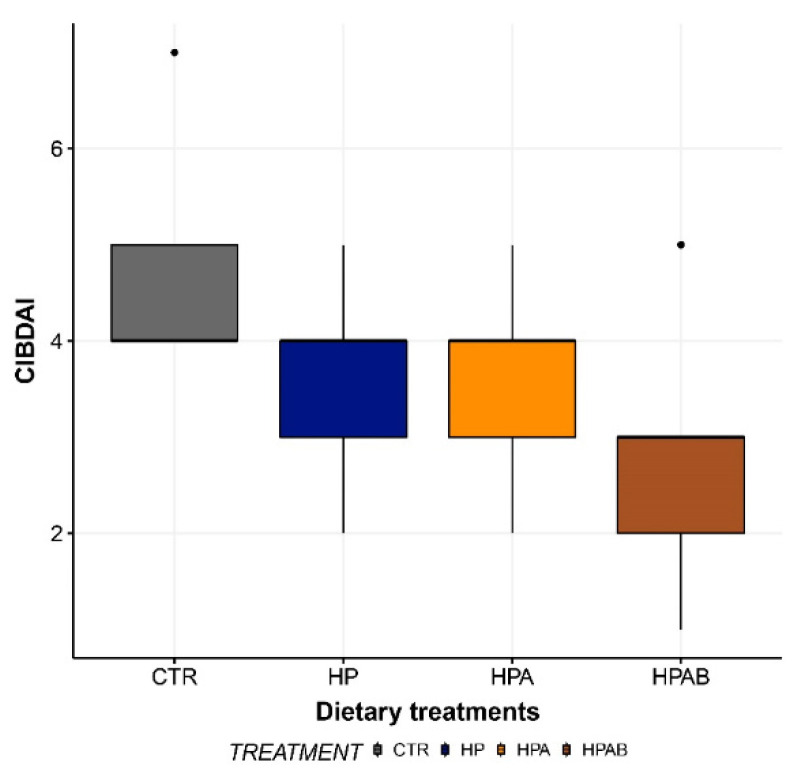
Canine Inflammatory Bowel Disease Activity Index (CIBDAI) score before the initiation of the study (CTR) and at the end of each dietary treatment. Data are represented as box and whisker plots showing median, quartiles, range, and outliers. CTR: Control diet; HP: Hydrolysed protein diet; HPA: *Ascophyllum nodosum*-supplemented HP diet; HPAB: *B. subtilis* C-3102 fortified HPA diet.

At the beginning of the study (d0), enrolled dogs had a median CIBDAI score of 4. While a progressive numerical reduction in median CIBDAI scores was observed throughout the length of the investigation, statistical significance was not reached in any of the comparisons made (Figure 2).

**Figure 3:**
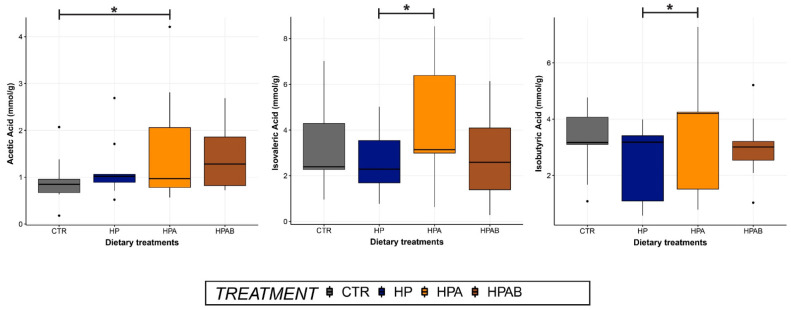
Faecal concentrations of acetic, isovaleric and isobutyric acids before the initiation of the study (CTR) and at the end of each dietary treatment. Data are represented as box and whisker plots showing median, quartiles, range, and outliers. Asterisks indicate statistical significance (*p* < 0.05). CTR: Control diet; HP: Hydrolysed protein diet; 2) HPA: *Ascophyllum nodosum*-supplemented HP diet; HPAB: *B. subtilis* C-3102 fortified HPA diet.

With respect to faecal fermentation products, a treatment effect was observed for acetic acid, which showed a significant increase (*p* < 0.05) in the HPA relative to CTR group. Besides, compared to the HP dietary treatment, HPA diet administration significantly augmented (*p* < 0.05) faecal concentrations of isovaleric and isobutyric acids (Figure 3). Furthermore, concentrations of butyric acid tended to be greater (*p* = 0.06) in the HPAB group. Ultimately, faecal indole content resulted to be greater (*p* < 0.05) in animals ageing 5 or less years of age (median (interquartile range): group 1 = 1.22 µg/g (1.47), group 2 = 0.62 µg/g (0.76)).

### 3.2. Faecal Microbiota Composition

#### 3.2.1. Faecal Microbiota Sequencing, OTU Identification and Annotation

**Figure 4:**
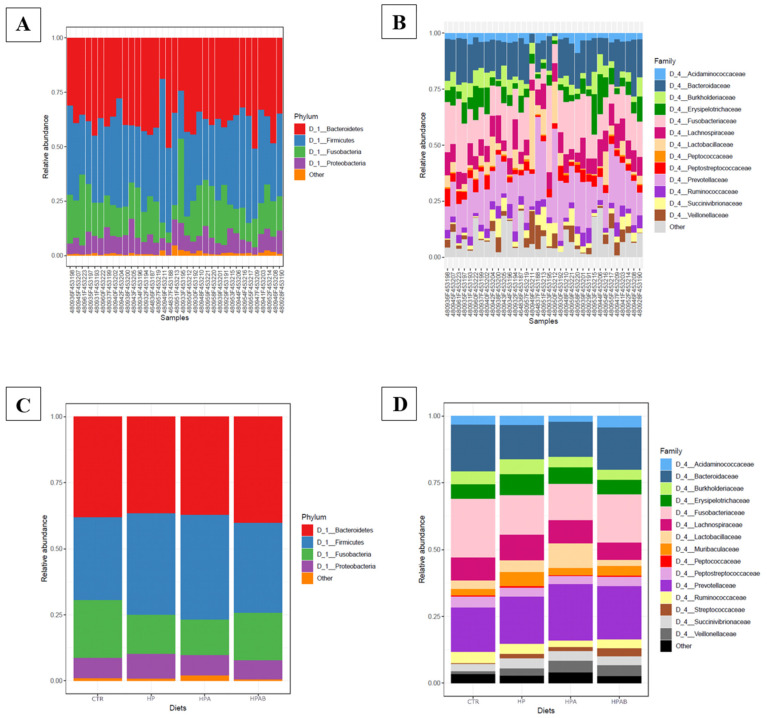
(**A**) Bar plot of the main phyla detected in faecal samples; (**B**) Bar plot of the main families detected in faecal samples; (**C**) Bar plot of the main phyla described for each dietary treatment; (**D**) Bar plot of the main families described for each dietary treatment.

Sequences from the whole sample set were successfully clustered into 393 OTUs. OTU counts per sample and OTU taxonomical assignments are available in Table S3. Overall, 338 of the 393 OTUs (86%) were assigned to a genus. The phyla Firmicutes (197/393) and Bacteroidetes (117/393) represented 50% and 29.7% of the OTUs, respectively. Within the phylum Firmicutes, 63.4% (125/197) of the OTUs were assigned to the order Clostridiales, 49.6% (62/125) to the family *Lachnospiraceae*, and 25.6% (32/125) to the family *Ruminococcaceae*. Within the phylum Bacteroidetes, 46.1% (54/117) of the OTUs belonged to the genus *Prevotella*. Other phyla were also represented, but in minor proportions (e.g., Proteobacteria: 7.3%, Fusobacteria: 6%, Epsilonbacteraeota: 3.30 %, Actinobacteria: 2%, Tenericutes: 0.7%, Spirochaetes: 0.5%, and Deferribacteres: 0.5%; Figure 4).

#### 3.2.2. Effects of Supplementation on Faecal Microbiota Composition and Diversity among Dietary Treatments

For all pairwise comparisons between dietary treatments, no significant differences were found in terms of faecal microbiota composition (Adonis test: *p* > 0.05). The same results were obtained for alpha diversity, beta diversity and observed microbial richness at OTU level (ANOVA test, *p* > 0.05).

**Figure 5:**
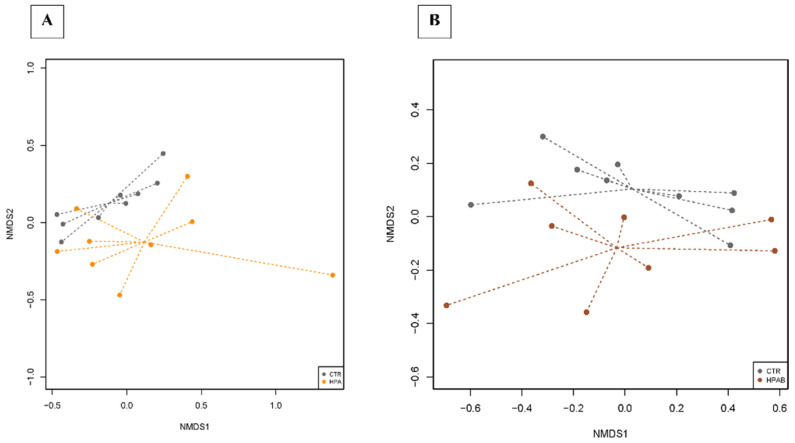
Dissimilarities in faecal microbiota composition represented by the non−metric multidimensional scaling (NMDS) ordination plot, with Bray−Curtis dissimilarity index calculated on unscaled OTU abundances. The centroids of each dietary treatment are features as the treatment name on the graph (‘env_fit’; Vegan R package). Faecal samples collected from CTR vs. HPA diets (**A**) and from CTR vs. HPAB diets were compared (**B**). Samples are coloured according to the dietary treatment: CTR (control diet, grey), HPA (hydrolysed protein diet + *Ascophyllum nodosum*; orange) and HPAB (hydrolysed protein diet + *Ascophyllum nodosum* + *B. subtilis* C-3102; brown). Larger filled circles indicate treatment centroids.

In the NMDS plots of the comparison between CTR vs. HPA and CTR vs. HPAB groups, the centroids of the dietary treatments appeared separated, even if resulting in a non-significant value (env_fit test, *p* > 0.05; Figure 5). The dietary treatments were used in the model of the differential analysis at the OTU level, and for the three comparisons (CTR vs. HP, CTR vs. HPA and CTR vs. HPAB) we described 66, 54 and 56 DA OTUs, respectively (Tables S4–S6). The faecal microbiota of dogs belonging to the HP dietary treatment was enriched with *Prevotella*, whereas the animals fed the HPA diet showed DA OTUs belonging mainly to the *Ruminococcaceae*, *Rikenellaceae,* and *Erysipelotrichaceae* families. In the analysis carried out between CTR vs. HPAB dietary treatments, instead, OTUs belonging to the *Enterococcus* and *Bacillus* genera were differentially abundant.

## 4. Discussion

Concerning the overall canine shelter population, a rather high (29.5%) number of dogs showing chronic GI signs, of which diarrhoea was the most common, was found. This was in agreement with another study [55], and not surprising, as it is likely to be consequential to environmental stressors (e.g., overcrowding, noise and limited physical activity) as well as infectious and parasitic diseases [56]. Notably, experimental evidence in murine models of human IBDs points out that stress and parasitic infestations might play a chief role in the pathogenesis of chronic inflammatory diseases of the gut [57,58]. Parasitological screening registered a prevalence rate of parasitic infestations (i.e., *Giardia duodenalis*, *Trichuris vulpis*, *Toxocara canis*, *Cryptosporidium* spp., *Ancylostoma* spp., and *Isospora* spp.) of 67% among canines with chronic GI issues.

The development of novel, less impactful management strategies is highly desired for chronic inflammatory diseases, which typically dictate either recurrent or life-long pharmacological intervention. In this context, there is a sound rationale for using functional foods based on seaweeds and/or probiotic microorganisms in dogs affected by food-unresponsive CIE. More specifically, the brown seaweed AN is known to possess anti-inflammatory properties thanks to its numerous nutraceutical compounds, first and foremost the sulphated fucans [59]. On the other hand, *B. subtilis* C-3102 can relieve intestinal inflammation via direct immune-modulation as well as microbiota-shaping properties, as demonstrated in vitro [60], and in vivo in the broiler chicken [61].

The CIBDAI score represents a reliable measure of inflammation-driven clinical signs in dogs with CE and is used worldwide to assess the initial response to treatment as well as long-term progress [40]. Although the administration of AN-derived fucoidans in a murine model of antibiotic-induced chronic colitis [62], and *B. subtilis* C-3102 in dogs with chronic diarrhoea [29], were found effective in improving clinical outcomes (e.g., weight gain, faecal quality), in the present study none of the treatments was able to produce a significant improvement in either faecal consistency or CIBDAI score. However, while not being statistically significant, it is worth reporting that the HPAB treatment was able to induce normalisation of stool quality in two dogs enrolled in the investigation. Potential contributors to the lack of therapeutic success may be a too short dietary treatment duration and/or an insufficient amount of agent added to the foods. In addition, as far as probiotic microorganisms are concerned, it must be stressed that their biological effects are likely strain-, disease-, and individual-specific [63]. It cannot be ruled out, therefore, that the use of another probiotic product, even at a lower dosage, might have produced better results.

Of the faecal fermentative end-products, acetic acid was most likely affected by the total dietary fibre content of the treatments (TDF CTR diet = 11.75%; TDF HP diet = 12.92%; TDF HPA diet = 18.02%), being the lowest in the CTR group, and peaking after HPA dietary intervention, where it reached statistical significance. It can be conjectured that the present finding reflected the inclusion of AN, a rich source of soluble fibres (e.g., alginic acid, laminarin, and sulphated polysaccharides) [12] in the HP diet, which stimulated acetogenesis within the gut microbiota, as already reported in humans and pre-ruminants [64,65]. In sharp contrast, a recent investigation carried out in healthy dogs failed to demonstrate any influence on faecal L-SCFA profile when intact AN was supplemented at a dose of 15 g/kg of diet (as is) for a maximum of 28 days [66]. It must be stressed, however, that the level of AN fortification used in the present study was higher. In addition, it was expected to have similar or greater amounts of acetic acid following HPAB diet administration, as *B. subtilis* is a well-established acetogenic bacterium [67]. A plausible explanation to this discrepancy may lie in the small sample size, which increased the influence of patient variability on statistical analyses. While its real contribution toward gut health is yet to be fully clarified in mammals, lines of evidence showed that acetic acid is involved in chief physiological processes, ranging from acidification of the gut lumen to regulation of mucosal blood flow and intestinal motility [68]. Furthermore, concentrations of all major L-SCFA were reported to be reduced in dogs with CIE relative to healthy controls [69], potentially underscoring a link between L-SCFA and canine chronic inflammatory enteropathies.

Isovaleric and isobutyric acids are two representatives of the B-SCFA, minor components of SCFA. These compounds, which are mainly produced via catabolism of branched-chain amino acids (i.e., valine, isoleucine, and leucine) [70], are generally viewed with a negative connotation, being a proxy marker of microbial putrefactive fermentations. Notwithstanding, B-SCFA may exert positive effects on the intestinal epithelium, via immune-modulation [71], control of trans-cellular ionic movements [72], and provision of an alternative colonic energy source (i.e., isobutyrate) in case of curtailed butyrate supply [73]. Our study observed a higher presence of isovaleric and isobutyric acids in faecal samples of dogs fed the HPA in comparison with HP diet. This result might be justified by the fact that the former dietary treatment had a greater fibre content which, aside from implementing saccharolysis, is acknowledged to reduce nutrient digestibility [74], therefore augmenting the magnitude of proteinaceous substrates being available for fermentation. Notably, concentrations of B-SCFA were lower following HPAB compared to HP dietary treatment. Previous research on various animal species and humans has shown that *B. subtilis* C-3102 is capable of reducing proteolysis, either through gut microbiota modulation [75,76], or by increasing apparent total tract digestibility of proteins [77]. Nevertheless, two independent studies in healthy adult dogs recorded no decline in faecal B-SCFA concentrations, in face of a reduction in gut ammonia, when administered a *B. subtilis* C-3102 fortified diet [27,28]. Yet, owing to the considerably lower dosage of probiotic used in those reports (1 × 10^9^ spores/kg of food as is), it cannot be ruled out that the numerically lower faecal content of isovaleric and isobutyric acids we registered in the HPAB group is ascribable to *B. subtilis* C-3102 supplementation.

Further, regarding the HPAB dietary treatment, a tendency for a higher faecal butyric acid content was recorded. Although there is lack of scientific evidence supporting its direct production of butyrate, *B. subtilis C-3102* is known to promote lactic acid synthesis by enhancing the growth of lactic acid bacteria [78]. Lactic acid, in turn, can be bio-transformed to different SCFA, first and foremost butyric acid, by cross-feeding [79,80,81]. This interpretation might also justify the fact that, in the present study, lactic acid did not increase after probiotic treatment.

Although it has been already shown in humans as well as in dogs that intestinal dysbiosis is related to reductions in tryptophan–indole pathways [6,82], our study found that age had an effect on faeces content of indole, which resulted to be higher in animals as old as 5 years (mean age of dogs completing the study: 5.1 ± 1.8 years; 3–8). This finding is not unprecedented: a human study described an inverse relationship between faecal indole synthesis and ageing, as a consequence of a progressive reduction in the expression of two gut microbial enzymes involved in tryptophan-to-indole metabolism (i.e., tryptophanase and tryptophan synthase) [83]. Even if indole has demonstrated to act as a co-carcinogen [84] and to be indirectly associated with chronic kidney disease [85], additional investigations have also revealed its positive impact over important homeostatic processes, such inflammation, intestinal barrier function, and response to xenobiotics [86]. Moreover, Foster et al. [87] demonstrated that an increased tryptophan catabolism limits the production of serotonin, a neurotransmitter that is essential for GI secretion, motility, and pain perception, which may be another mechanism through which tryptophan affects GI health.

Analysis of the faecal microbiota composition showed that Firmicutes and Bacteroidetes were the major phyla in our dataset. In several studies, alterations of specific gut bacterial groups between healthy canines and dogs with acute diarrhoea and CIE have been identified. As a hallmark of dysbiosis, an increased abundance of Proteobacteria (especially Gammaproteobacteria) and a decrease in Firmicutes (especially *Faecalibacterium*, *Ruminococcus* and *Blautia*) is generally observed in CIE dogs, relative to healthy controls [5]. With that being said, numbers of Firmicutes usually still remains higher than those of Proteobacteria within the faecal microbiota of dogs with CIE [88].

Bioinformatics analyses carried out on bacterial 16S rRNA gene sequences revealed no differences in microbial diversity indices (i.e., alpha diversity, beta diversity and microbial richness) among groups. However, the graphical representation of dissimilarities in faecal microbiota composition by NMDS ordination plot, showed a divergence of centroids when comparing bacterial communities from CTR vs. HPA groups. The diversity became more appreciable when faecal samples from CTR vs. HPAB groups were compared. Again, a larger sample size would have diminished the impact of inter-individual variability in gut microbiota composition on biostatical analyses, plausibly yielding statistical significance.

Differential analysis of the relative abundance of bacteria at the OTUs level showed the genus *Prevotella* to be less abundant at d0 compared to HP dietary treatment. It has been reported that bacterial groups belonging to the *Prevotellaceae* family are underrepresented in dogs with idiopathic inflammatory bowel disease (IBD) relative to healthy control dogs [89]. Members of the *Prevotella* genus are regarded to be L-SCFA producers, as they can ferment both xylan and cellulose through carbohydrate-active enzymes (e.g., xylanase, carboxymethylcellulase, and endoglucanase) [90]. It is highly likely that the higher abundance of the *Prevotella* genus we observed after the administration of the HP diet is consequential to the differences in macronutrient content between the CTR and HP diets, the latter being richer in dietary fibre, as also showed in other reports [91]. Instead, the HPA group was characterised by increased numbers of *Ruminococcaceae* and *Rikenellaceae*, two other taxa involved in the degradation of carbohydrates and production of volatile fatty acids [92,93]. This datum is in line with results of previous microbiological studies in mice fed fucoidans from AN [62,94], and corroborates the higher faecal amounts of acetic acid found following AN supplementation in the diet. Likewise, the augmented concentrations of isovalerate and isobutyrate in faeces upon administration of the HPA diet was accompanied by a higher abundance of unknown genera belonging to the *Erysipelotrichaceae* family, which has been negatively correlated with crude protein digestibility and positively correlated with metabolites of protein digestion in dogs [95]. Lastly, consumption of the HPAB diet was associated with greater numbers of *Enterococcus* and *Bacillus*, the latter being absent in the previous diets, and thus plausibly mirroring the probiotic supplementation itself. Our finding of increased abundance of faecal enterococci upon *B. subtilis* C-3102 ingestion is in disagreement with data recorded by de Lima et al. [28]. While some *Enterococcus* strains may possess pathogenic traits, others have shown beneficial effects to the gut microbiota, for example via the production of bacteriocins [96]. Furthermore, even though they are not classified as butyrogenic bacteria, members of the *Enterococcus* genus are known producers of lactic acid, which, as previously mentioned, could potentially boost intestinal butyrate formation by cross-feeding.

Several limitations to this pilot study need to be acknowledged, some of which pertain to the diagnostic exclusion protocol, whereas others to the dietary trial itself. With respect to the former, our strategy of excluding infected animals from the diagnostic work-up due to the risk of re-infestation after parasiticidal therapy might have caused a loss of other CIE cases within the study population, being intestinal parasitoses of common occurrence in primary CIE-affected dogs [97]. Furthermore, the lack of a complete dietary history in all patients belonging to the study group might have contributed to CIE phenotype misclassification. In order to compensate for this, it was decided to conduct the dietary trial with a fit-for-purpose hydrolysed animal protein diet, characterised by a simple ingredient composition. Besides, it is unlikely that antibiotic-responders would have had FRE. Moreover, animals diagnosed with IRE had been fed a variety of commercial foods, including hydrolysed diets, prior to study initiation without showing any clinical improvement. Due to legal and logistical constraints, it was only possible to perform instrumental tests (i.e., abdominal ultrasound and digestive endoscopy) as well as histopathology of endoscopically retrieved biopsies on animals failing to respond to tylosin administration. This flaw might have precluded the identification of potential causes of secondary ARE, such as partial intestinal obstruction [98]. However, none of the ARE patients presented with an alternation of constipation and diarrhoea, which is expected to be seen in such cases. Lastly, it is unlikely for stress diarrhoea to be accountable for reported clinical signs in the study group due to the fact that all the patients were admitted to the shelter several months prior to the beginning of the investigation, and were regarded as well-accustomed to their living environment on the basis of behavioural evaluations.

Concerning the dietary trial design, some additional pitfalls are worthwhile of being discussed. First, since 3 months before study initiation all the enrolled animals were preliminarily subjected to antibiotic trial with tylosin, which may cause long-term alterations of the canine faecal microbiota [99], it cannot be ruled out that the final results were affected by the diagnostic procedure. In addition, the inability to carry out multiple coprological tests all along the study period did not allow to completely exclude new parasitic infestations within the study population. Finally, it must be underscored that the small size of sample, coupled with its heterogeneity in terms of CIE clinical phenotypes (ARE, IRE), likely reduced the power of the statistical analysis.

## 5. Conclusions

In conclusion, this pilot study failed to prove the efficacy of AN, with or without *B. subtilis C-3102* supplementation, in terms of clinical outcome and microbial richness parameters in dogs with CIE. Notwithstanding, the increase in faecal acetic acid concentrations, as well as a higher abundance of beneficial bacteria, might indicate favourable effects deriving from the administration of the tested treatments on gut health. Owing to the main limitations of the current investigation, it is important to point out that further research is urgently needed in order to confirm the importance of nutritional alternatives to classic pharmacotherapy in the management of CIE.

## Figures and Tables

**Table 1:** Ingredients (% as fed basis) and chemical composition of the extruded dog food used for the dietary trial.

Raw Materials (%)		Nutrients	g/100 g	g/Mcal
		Analysed		
Protein pea seeds	53.3	DM	92.71	252.27
Potato starch	20.0	CP	20.09	54.67
Pork protein hydrolysate	11.3	EE	12.92	35.16
Pork fat	6.2	Ashes	7.44	20.25
Corn starch	3.5	CF	3.2	8.6
Calcium carbonate	1.8	NDF	11.8	32.2
Additives *	1.7	ADF	3.2	8.7
Pure cellulose	1.0	ADL	0.3	0.8
Anhydrous di-calcium phosphate	1.0	TDF	13.0	35.4
Halite salt	0.2	Calculated		
			
		ME (kcal/kg)	3675.0	

Nutritional Additives *: Vitamin A: 13,400 IU/kg, Vitamin D: 1200 IU/kg, Vitamin E: 800 mg/kg; Vitamin B1: 4.6 mg/kg; Vitamin B2: 3.5 mg/kg; Vitamin B5: 2.2 mg/kg; Vitamin B6: 3 mg/kg; Vitamin B12:0.07 mg/kg; Nicotinic acid: 17 mg/kg; Folic acid: 0.22 mg/kg; Vitamin C: 240 mg/kg; Biotin: 0.13 mg/kg; Choline: 280 mg/kg; Zinc chelate of amino acids hydrate 130 mg/kg (zinc 13 mg/kg), Potassium iodide: 0.73 mg/kg (0.56 mg/kg iodine). Technological additives *: Clinoptilolite: 80 g/kg; BHA + BHT: 16 mg/kg. DM: dry matter; CP: crude protein; EE: ether extracts; CF: crude fibre; NDF: neutral detergent fibre; ADF: acid detergent fibre; ADL: acid detergent lignin; TDF: total dietary fibre; ME: metabolisable energy.

## Data Availability

The raw sequencing data has been submitted to NCBI’s Sequence Read Archive (SRA) repository (BioProject: PRJNA692677; Biosample: SUB8781999).

## Supplementary Materials

The following are available online at https://www.mdpi.com/article/10.3390/ani11123417/s1, Figure S1. Drop-in/drop-off flowchart of the diagnostic exclusion protocol. Table S1. Summary of the metadata of the 9 animals involved in the study. Table S2. Statistical summary of data regarding faecal metabolites. Table S3. OTU taxonomical assignments and OTU counts in each sample of the whole dataset. Table S4. Differentially abundant OTUs when comparing the faecal microbiota composition between CTR and HP diets. Table S5. Differentially abundant OTUs when comparing the faecal microbiota composition between CTR and HPA diets. Table S6. Differentially abundant OTUs when comparing the faecal microbiota composition between CTR and HPAB diets.
