# Supplementary material for: The Use of Ascophyllum nodosum and Bacillus subtilis C-3102 in the Management of Canine Chronic Inflammatory Enteropathy: A Pilot Study
Source: Animals (Basel). 2021 Nov 30;11(12):3417. doi: 10.3390/ani11123417 (PMC8697907; doi:10.3390/ani11123417)
Supplement: Supplementary file 1 [file animals-11-03417-s001.zip › supplementary_materials/Table_S1.pdf]

Table S1. Summary of the metadata of the 9 animals involved in the study.

									Timepoint d0 – Diet CTR		Timepoint d30 – Diet HP		Timepoint d60 – Diet HPA		Timepoint d90 – Diet HPAB	
**Dog ID**	**Breed**	**Sex**	**Age**	**Neuter status**	**Weight**	**BCS**	**Histopathological diagnosis**	**Disease subtype**	**Sample ID**	**CIBDAI**	**Sample ID**	**CIBDAI**	**Sample ID**	**CIBDAI**	**Sample ID**	**CIBDAI**
1	Crossbreed	Male	7	Castrated	34	5	Moderate lymphocytic-plasmacytic enteritis	IRE	480928F453190	5	480937F453199	3	480946F453208	4	464836F453187	1
2	Pit Bull	Male	3	Castrated	18.35	4	-	ARE	480929F453191	4	480938F453200	4	480947F453209	3	464837F453188	2
3	Crossbreed	Male	5	Castrated	29.8	7	-	ARE	480930F453192	4	480939F453201	3	480948F453210	2	480955F453217	2
4	Italian Corso Dog	Male	5	Castrated	38	5	Moderate lymphocytic-plasmacytic duodenitis	IRE	480931F453193	7	480940F453202	5	480949F453211	4	480956F453218	3
5	Crossbreed	Male	3	Castrated	30	6	-	ARE	480932F453194	4	480941F453203	2	480950F453212	2	480957F453219	3
6	Crossbreed	Female	9	Spayed	29.4	5	-	ARE	480933F453195	4	480942F453204	2	480951F453213	3	480958F453220	3
7	American Bulldog	Male	6	Castrated	31.1	5	-	ARE	480934F453196	5	480943F453205	4	480952F453214	5	480959F453221	5
8	Italian Corso Dog	Male	6	Castrated	40.6	4	-	ARE	480935F453197	4	480944F453206	4	480953F453215	5	480960F453222	3
9	Crossbreed	Male	6	Castrated	25.9	5	Severe lymphocytic-plasmacytic duodenitis	IRE	480936F453198	4	480945F453207	4	480954F453216	4	480961F453223	5
