# Supplementary material for: The Use of Ascophyllum nodosum and Bacillus subtilis C-3102 in the Management of Canine Chronic Inflammatory Enteropathy: A Pilot Study
Source: Animals (Basel). 2021 Nov 30;11(12):3417. doi: 10.3390/ani11123417 (PMC8697907; doi:10.3390/ani11123417)
Supplement: Supplementary file 1 [file animals-11-03417-s001.zip › supplementary_materials/Table_S2.pdf]

Table S2. Statistical summary of data regarding faecal metabolites.

Variable	Dietary treatment															
	CTR				HP				HPA				HPAB			
	Min	Max	Median	Interquartile Range	Min	Max	Median	Interquartile Range	Min	Max	Median	Interquartile Range	Min	Max	Median	Interquartile Range
CIBDAI	4.0	7.0	4.0	1	2.0	5.0	4.0	1	2	5	4	1	1	5	3	1
Acetic Acid (mmol/g)	0.18	2.07	0.85	0.29	0.52	2.69	1.02	0.17	0.57	4.21	0.97	1.28	0.72	2.69	1.28	1.04
Acetone (mmol/g)	0.00	1.01	0.01	0.01	0.00	0.09	0.01	0.00	0.00	1.03	0.01	0.02	0.00	0.86	0.01	0.02
Butyric Acid (mmol/g)	0.37	3.07	0.92	0.64	0.37	1.52	0.71	1.01	0.52	2.37	1.06	0.28	0.37	4.05	1.06	0.82
Formic Acid (mmol/g)	0.05	1.39	0.75	0.85	0.02	1.52	0.72	0.67	0.02	2.09	0.63	0.85	0.09	1.06	0.28	0.57
Indole (µg/g)	0.15	3.18	0.80	0.54	0.03	2.06	0.78	0.81	0.19	3.41	0.97	0.55	0.17	2.08	0.52	0.8
Isobutyric Acid (mmol/g)	1.08	4.77	3.17	0.97	0.57	3.99	3.18	2.32	0.78	7.28	4.21	2.74	1.03	5.21	3.01	0.67
Isovaleric Acid (mmol/g)	0.96	7.02	2.39	2.02	0.77	5.02	2.29	1.85	0.63	8.54	3.14	3.4	0.28	6.14	2.59	2.7
Lactic Acid (mmol/g)	0.06	0.28	0.21	0.06	0.05	0.81	0.21	0.05	0.05	0.63	0.28	0.2	0.02	0.89	0.5	0.38
Phenol (µg/g)	0.15	3.05	0.72	0.46	0.03	3.21	0.36	1.5	0.13	3.04	0.64	1.91	0.13	3.10	0.92	0.84
Propionic Acid (mmol/g)	0.09	2.26	0.51	0.58	0.07	1.05	0.52	0.03	0.03	1.09	0.28	0.57	0.02	2.29	0.51	0.58
Skatole (µg/g)	0.16	2.16	0.52	0.78	0.24	2.01	0.98	0.34	0.21	2.16	0.89	0.72	0.19	2.39	1.22	1
Valeric Acid (mmol/g)	0.52	5.91	0.72	0.26	0.42	4.28	0.98	0.29	0.50	2.29	0.93	0.39	0.45	1.06	0.85	0.19

CTR: Control diet; HP: Hydrolysed protein diet; 2) HPA: *Ascophyllum nodosum*-supplemented HP diet; HPAB: *B. subtilis* C-3102 fortified HPA diet.
