# Supplementary material for: The Use of Ascophyllum nodosum and Bacillus subtilis C-3102 in the Management of Canine Chronic Inflammatory Enteropathy: A Pilot Study
Source: Animals (Basel). 2021 Nov 30;11(12):3417. doi: 10.3390/ani11123417 (PMC8697907; doi:10.3390/ani11123417)

Drop-in/drop-off flowchart

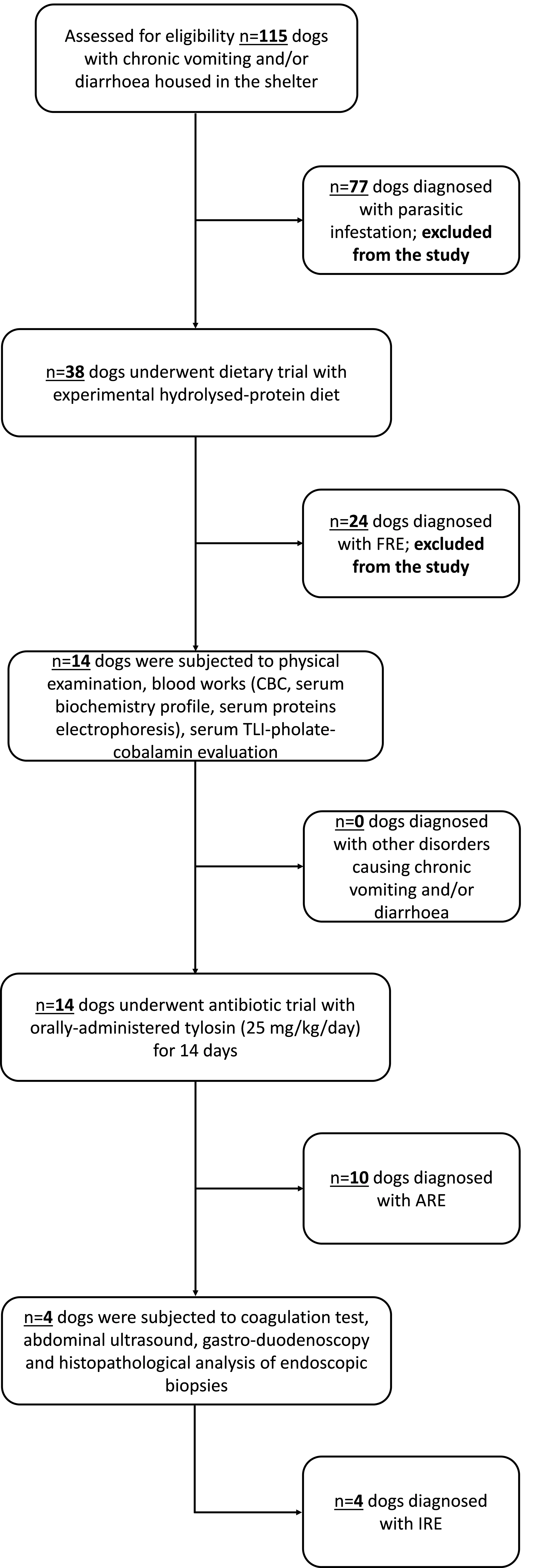

Supplement: Supplementary file 1 [file Figure S1.pdf › supplementary_materials/Figure S1.pdf]
